# McImpute: Matrix Completion Based Imputation for Single Cell RNA-seq Data

**DOI:** 10.3389/fgene.2019.00009

**Published:** 2019-01-29

**Authors:** Aanchal Mongia, Debarka Sengupta, Angshul Majumdar

**Affiliations:** ^1^Department of Computer Science and Engineering, Indraprastha Institute of Information Technology Delhi, New Delhi, India; ^2^Center for Computational Biology, Indraprastha Institute of Information Technology Delhi, New Delhi, India; ^3^Department of Electronics and Communications Engineering, Indraprastha Institute of Information Technology Delhi, New Delhi, India

**Keywords:** scRNA-seq, dropouts, imputation, matrix completion, Nuclear norm minization

## Abstract

**Motivation:** Single-cell RNA sequencing has been proved to be revolutionary for its potential of zooming into complex biological systems. Genome-wide expression analysis at single-cell resolution provides a window into dynamics of cellular phenotypes. This facilitates the characterization of transcriptional heterogeneity in normal and diseased tissues under various conditions. It also sheds light on the development or emergence of specific cell populations and phenotypes. However, owing to the paucity of input RNA, a typical single cell RNA sequencing data features a high number of dropout events where transcripts fail to get amplified.

**Results:** We introduce mcImpute, a low-rank matrix completion based technique to impute dropouts in single cell expression data. On a number of real datasets, application of mcImpute yields significant improvements in the separation of true zeros from dropouts, cell-clustering, differential expression analysis, cell type separability, the performance of dimensionality reduction techniques for cell visualization, and gene distribution.

**Availability and Implementation:** https://github.com/aanchalMongia/McImpute_scRNAseq

## 1. Background and Introduction

In contrast to traditional bulk population-based expression studies, single-cell transcriptomics provides more precise insights into the functioning of individual cells. Over the past few years, this powerful tool has brought in transformative changes in the conduct of functional biology (Wagner et al., 2016). With single-cell RNA sequencing (scRNA-seq) we are now able to discover subtypes within seemingly similar cells. This is particularly advantageous for characterizing cancer heterogeneity (Patel et al., 2014; Tirosh et al., 2016), identification of new rare cell type and understanding the dynamics of transcriptional changes during development (Tang et al., 2010; Yan et al., 2013; Biase et al., 2014).

Despite all the goodness, scRNA-seq technologies suffer from a number of sources of technical noise. Most important of these is insufficient input RNA. Due to small quantities transcripts are frequently missed during the reverse transcription step. As a direct consequence, these transcripts are not detected during the sequencing step (Kharchenko et al., 2014). Often times the lowly expressed genes are the worst hit. Excluding these genes from the analysis may not be the best solution as many of the transcription factors and cell surface markers are sacrificed in this process (van Dijk et al., 2017). Added to that, variability in dropout rate across individual cells or cell types works as a confounding factor for a number of downstream analyses (Sengupta et al., 2016; Li et al., 2017). Hicks et al. (2015) showed, on a number of scRNA-seq datasets, that the first principal components highly correlate with the proportion of dropouts across individual transcriptomes. In summary, there is a standing need for efficient methods to impute scRNA-seq datasets.

**Figure 1:**
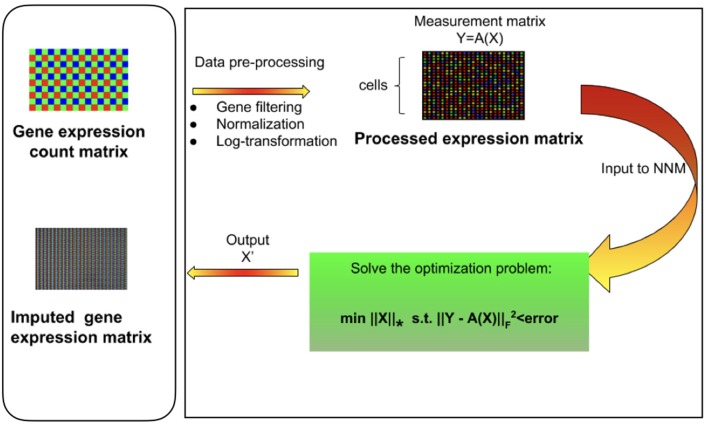
Overview of mcImpute framework for imputing single-cell RNA sequencing data. Raw read counts were filtered for significantly expressed genes and then normalized by Library size. Then, the expression data was Log_2_ transformed (after adding a pseudo-count of 1). This pre-processed expression matrix (Y) is treated as the measurement/observation matrix (and fed as input to Nuclear-norm minimization algorithm) from which the gene expressions of the complete matrix (X) need to be recovered by solving the non-convex optimization problem. The objective function minimizes the nuclear norm of expression matrix and satisfies the constraint Y = A(X) with minimum error; where Y is the sampled version of the complete expression matrix X and A is the sub-sampling operator.

Very recently, efforts have been made to devise imputation techniques for scRNA-seq data (Table S6). Most notable of among these are MAGIC (van Dijk et al., 2017), scImpute (Li and Li, 2018), and drImpute (Kwak et al., 2017). MAGIC uses a neighborhood based heuristic to infer the missing values based on the idea of heat diffusion, altering all gene expression levels including the ones not affected by dropouts. On the other hand, scImpute first estimates which values are affected by dropouts based on Gamma-Normal mixture model and then fills the dropout values in a cell by borrowing information of the same gene in other similar cells, which are selected based on the genes unlikely affected by dropout events. The overall performance of scImpute has been shown to be superior to MAGIC. Parametric modeling of single-cell expression is challenging due to our lack of knowledge about possible sources of technical noise and biases (Sengupta et al., 2016). Moreover, there is a clear lack of consensus about the choice of the probability density function. Another method, Drimpute, repeatedly identifies similar cells based on clustering and performs imputation multiple times by averaging the expression values from similar cells, followed by averaging multiple estimations for final imputation. We propose mcImpute (Figure 1), an imputation algorithm for scRNA-seq data which models gene expression as a low-rank matrix and sprouts in values in place of dropouts in the process of recovering the full gene expression data from sparse single-cell data. This is done by applying soft-thresholding iteratively on singular values of scRNA-seq data. One of the salient features of mcImpute is that it does not assume any distribution for gene expression.

We first evaluate the performance of mcImpute in separating “true zero" counts from dropouts on single-cell data of myoblasts (Trapnell et al., 2014) (We call it Trapnell dataset). On the same dataset, we assess the impact of imputation on differential genes prediction. We further investigate mcImpute's ability to recover artificially planted missing values in a single cell expression matrix of mouse neurons (Usoskin et al., 2015). Accurate imputation should enhance cell type identity i.e., the transcriptomic similarity between cells of identical type. We, therefore, quantify cell type separability as a metric and assess its improvement. In addition to these, we also test the impact of imputation on cell clustering. Four independent datasets Zeisel (Zeisel et al., 2015), Jurkat-293T (Zheng et al., 2017), Preimplantation (Yan et al., 2013) and Usoskin (Usoskin et al., 2015), for which cell type annotations are available and another dataset, Trapnell et al. (2014) for which bulk RNA-seq data has been provided (required for validation of differential genes prediction and separation of “true zeros" from dropouts), are used for this purpose. McImpute clearly serves as a crucial tool in the scRNA-seq pipeline by significantly improving all the above-mentioned metrics and outperforming the state-of-the-art imputation methods in the majority of experimental conditions.

With the advent of droplet-based, high-throughput technologies (Macosko et al., 2015; Zheng et al., 2017), library depth is being compromised to curb the sequencing cost. As a result, scRNA-seq datasets are being produced with an extremely high number of dropouts. We believe that mcImpute's great performance, will provide an adequate solution for the dropouts problem.

## 2. Results

We performed computational experiments to evaluate the efficacy of our proposed imputation technique comparing mcImpute with a number of existing imputation methods for single cell RNA data: scImpute, drImpute, and MAGIC.

### 2.1. Dropouts vs. True Zeros

The inflated number of zero counts in scRNA-seq data could either be biologically driven or due to lack of measurement sensitivity in sequencing. The transcript which is not detected because of failing to get amplified in the sequencing step essentially corresponds to a “false zero" in the finally observed count data and needs to be imputed. A reasonable imputation strategy which has this discriminating property should keep the “true zero" counts (where the genes are truly expressed and have no transcripts from the beginning) untouched, while at the same time attempt to recover the dropouts.

The goodness of an imputation strategy can be formally confirmed by observing two factors. First, whether the imputation method is able to impute the true zero counts in the expression data as is or not; Second, if it can fill-in the dropouts with biologically meaningful expression counts or not; showing an increasing difference between the zero counts observed in unimputed data and the imputed one with expression amplification.

We investigate the performance of mcImpute in distinguishing “true zero" counts from dropouts on Trapnell data (Trapnell et al., 2014), for which the bulk-counterpart was available and hence, we could pull out low-to-medium expression genes from the corresponding bulk data for validation. Of note, to differentiate between the “true" and “false" zeros, we have used the matched bulk-expression profiles; as it is a well-known fact that bulk-RNA seq data has limited or no dropouts events as the corresponding experiments involve millions of cells. The fraction of zero counts was observed for genes with expression ranging from zero to 500 for unimputed and imputed gene-expression data. It should be noted that an imputed count value ranging from 0 to 0.5 is taken as an imputed zero, rendering minor flexibility to all imputation techniques.

Given the nature of this analysis, gene filtering in single cell expressions has been skipped. DrImpute could not be taken into account since we could not programmatically mute the gene filtering step in its pipeline.

**Figure 2:**
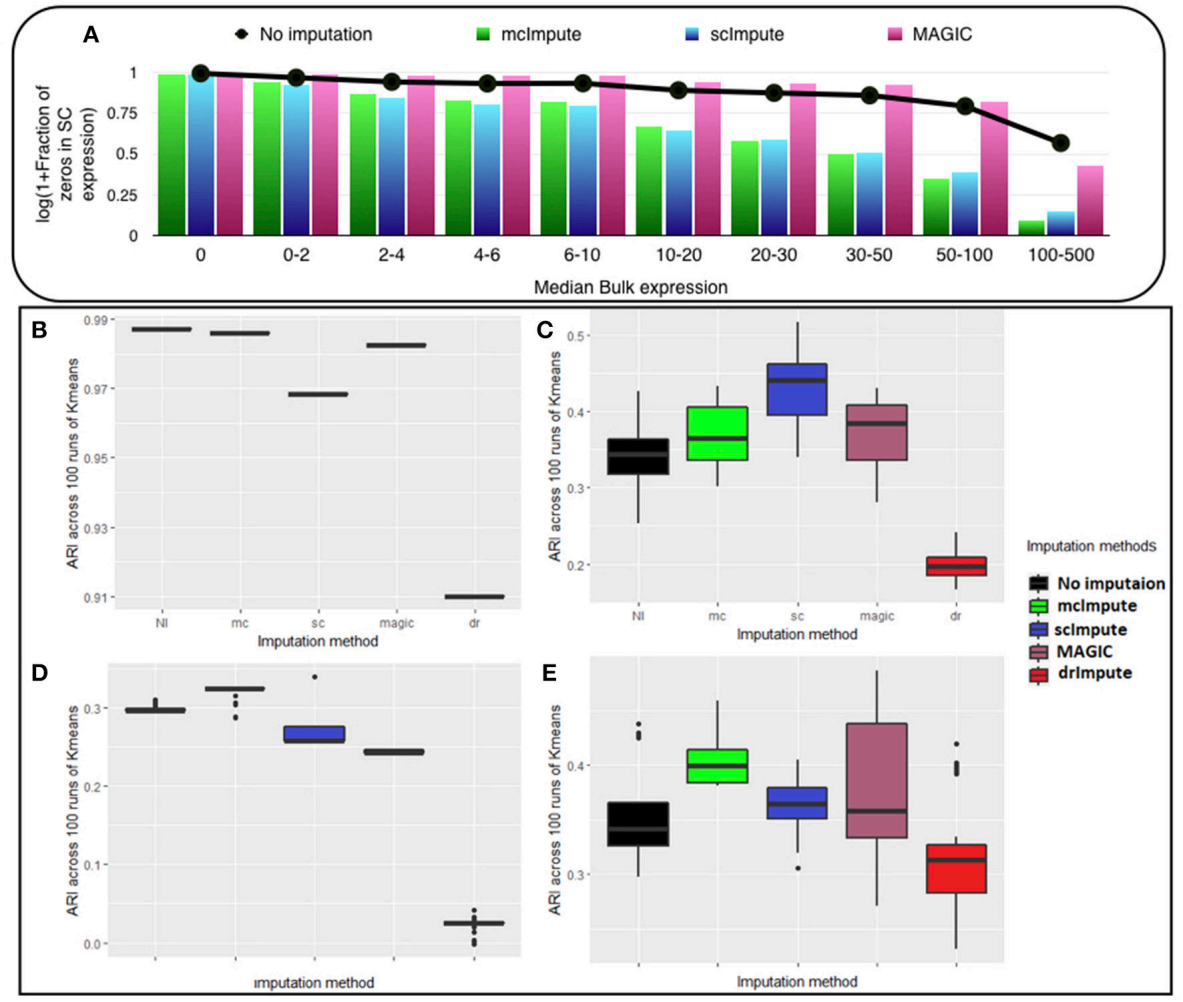
McImpute shows remarkable improvement in separation of “true zeros" from dropouts and clustering of single cells **(A)** Separation of “true zeros" from dropouts: plot showing fraction of zero counts (values between 0 and 0.5) in single cell expression matrix against the median bulk expression. The genes are divided into 10 bins based on median bulk genes expression (first bin corresponds to zero expression genes) **(B–E)** Boxplots showing the distribution of ARI calculated on 100 runs of k-means clustering algorithm on first two principal components of single cell expression matrix for datasets **(B)** Jurkat-293T **(C)** Preimplantation **(D)** Usoskin, and **(E)** Zeisel.

We observe (Figure 2A, Table S1) that with low expression genes, all imputation strategies successfully impute the “true zeros” while, as the gene expression amplifies, un-imputed matrix still exhibits large fraction of zeros, which essentially correspond to dropouts and only mcImpute and scImpute are able to curtail the fraction of zeros, thus recovering the dropouts back. As can be observed, MAGIC although successfully imputes the “true zeros"; it fails to recover most of the dropouts in the expression data.

### 2.2. Improvement in Clustering Accuracy

A correct interpretation of single-cell expression data is contingent on the accurate delineation of cell types. Bewildering level of dropouts in scRNA-seq data often introduces batch effect, which inevitably traps the clustering algorithm. A reasonable imputation strategy should fix these issues to a great extent. In a controlled setting, we, therefore, examined if the proposed method enhanced clustering outcomes. For this, we ran *K*-means on first 2 principal component genes of log-transformed expression profiles featured in each dataset (Figure S5). Since the prediction from this clustering algorithm tends to change with the choice of initial centroids, which are chosen at random, we analyze the results on 100 runs of k-means to get reliable and robust results. We set the number of annotated cell types as the value of *K* for every data. Adjusted Rand Index (ARI) was used to measure the correspondence between the clusters and the prior annotations.

McImpute based re-estimation best separates the four groups of mouse neural single cells from Usoskin dataset and brain cells from Zeisel dataset, and clearly shows comparable improvement on other datasets too (Figures 2B–E, Table S2). The striking difference between Jurkat and 293T cells made them trivially separable through clustering, leading to same ARI across all 100 runs. Still, mcImpute was able to better maintain the ARI in comparison to other imputation methods.

### 2.3. Matrix Recovery

In this set of experiments, we study the choice of matrix completion algorithm – matrix factorization (MF) or nuclear norm minimization (NNM). Both the algorithms have been explained in section Materials and Methods.

**Figure 3:**
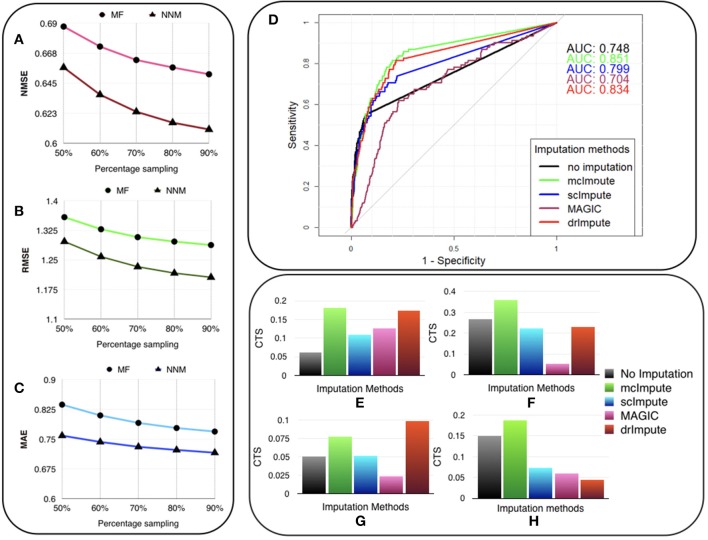
McImpute recovers the original data from their masked version with low error, performs best in prediction of differentially expressed genes and significantly improves CTS score. Variation of **(A)** NMSE, **(B)** RMSE, and **(C)** MAE with sampling ratio using MF (Matrix factorization) and NNM (Nuclear norm minimization) on Usoskin dataset showing NNM performing better than MF algorithm. **(D)** ROC curve showing the agreement between DE genes predicted from scRNA and matching bulk RNA-Seq data (Trapnell et al., 2014). DE calls were made on expression matrix imputed using edgeR. **(E–H)** 2D-Axis bar plot depicting improvement in Cell type separabilities between **(E)** Jurkat and 293T cells from Jurkat-293T dataset; **(F)** 8cell and BXC cell types from Preimplantation dataset; **(G)** NP and NF cells from Usoskin dataset; and **(H)** S1pyramidal and Ependymal from Zeisel dataset . Refer Table S4 for absolute values.

The experiments are carried out on the processed Usoskin dataset (Usoskin et al., 2015). We artificially removed some counts at random (sub-sampling) in the data to mimic dropout cases and used our algorithms (MF and NNM) to impute the missing values. (Figures 3A–C) and Table S3 show the variation of Normalized Mean Squared Error (NMSE), Root Mean Squared Error (RMSE) and Mean Absolute Error (MAE) to compare our two methods for different sub-sampling ratios. This is the standard procedure to compare matrix completion algorithms (Keshavan et al., 2010; Marjanovic and Solo, 2012).

We are showing the results for Usoskin dataset, but we have carried out the same analysis for other datasets and the conclusion remained the same. We find that the nuclear norm minimization (NNM) method performs slightly better than the matrix factorization (MF) technique; so we have used NNM as the workhorse algorithm behind mcImpute.

### 2.4. Improved Differential Genes Prediction

Optimal imputation of expression data should improve the accuracy of differential expression (DE) analysis. It is a standard practice to benchmark DE calls made on scRNA-Seq data against calls made on their matching bulk counterparts (Kharchenko et al., 2014). To this end, we used a dataset of myoblasts, for which matching bulk RNA-Seq data were also available (Trapnell et al., 2014). For simplicity, this dataset has been referred to as the Trapnell dataset. DE and non-DE genes were identified using edgeR (Zhou et al., 2014) package in R.

We used the standard Wilcoxon Rank-Sum test for identifying differentially expressed genes from matrices imputed by various methods. Congruence between bulk and single cell-based DE calls were summarized using the Area Under the Curve (AUC) values yielded from the Receiver Operating Characteristic (ROC) curves (Figure 3D). Among all the methods mcImpute performed best with an AUC of 0.85.

For each method, the AUC value was computed on the identical set of ground truth genes. We had to make an exception only for drImpute as it applies the filter to prune genes in its pipeline. Hence AUC value for drImpute was computed based on a smaller set of ground truth genes.

### 2.5. Improvement in Cell Type Separability

Downstream analysis becomes much easier if expression similarities between cells of identical type are considerably higher than that of cells coming from different subpopulations. To this end, we define the cell-type separability score as follows:

For any two cell groups, we first find the median of Spearman correlation values computed for each possible pair of cells within their respective groups. We call the average of the median correlation values the intra-cell type scatter. On the other hand, inter-cell type scatter is defined as the median of Spearman correlation values computed for pairs such that in each pair, cells belong to two different groups. The difference between the intra-cell scatter and inter-cell type scatter is termed as the cell-type separability (CTS) score. We computed CTS scores for two sample cell-type pairs from each dataset. In more than 80 % (13 out of 16) of test cases, mcImpute yielded significantly better CS values (Figures 3E–H, Table S4).

### 2.6. Cell Visualization

**Figure 4:**
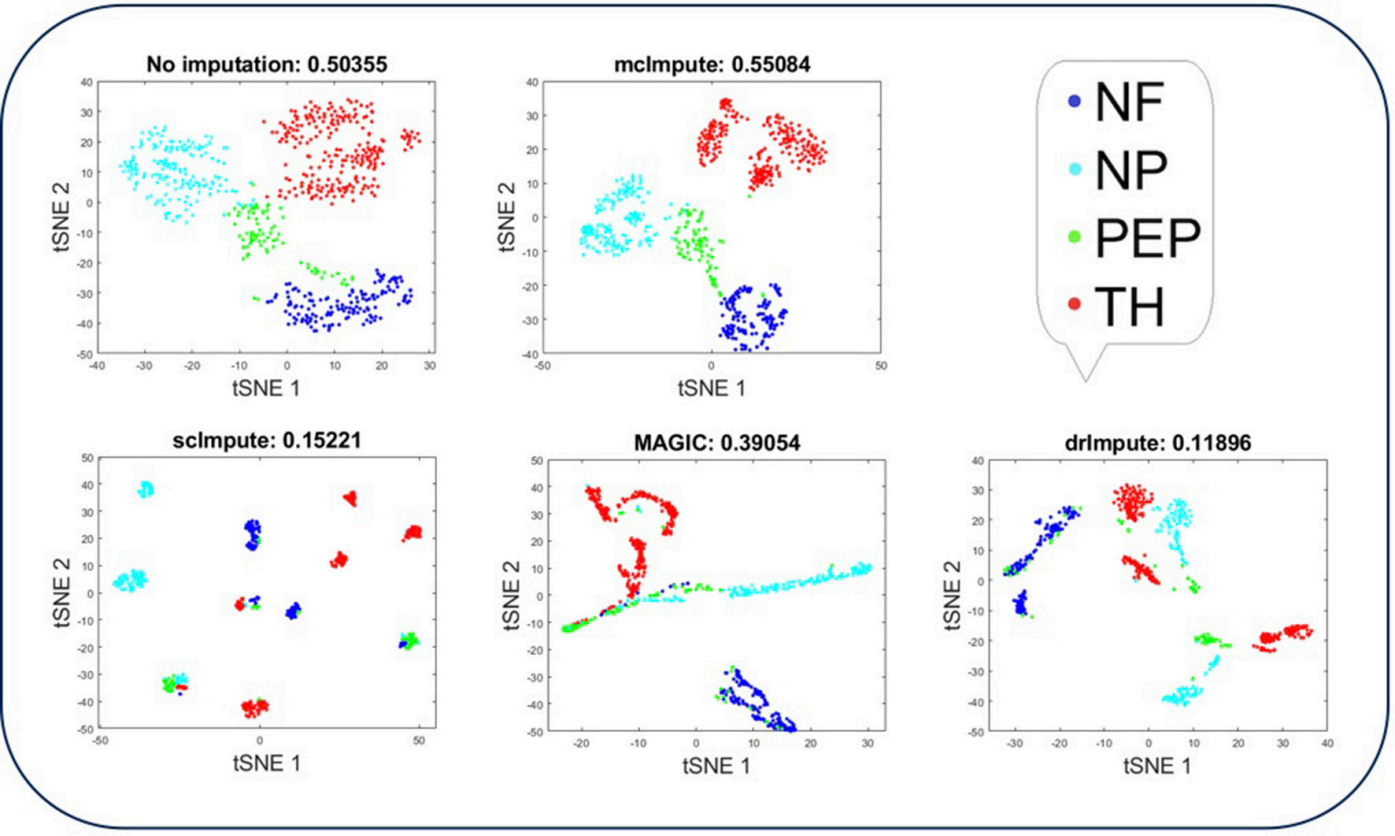
Plot showing t-SNE visualization and average silhouette values for Usoskin dataset before and after imputation. McImpute improves the visual distinguishability the most for all groups of mouse neural single cells amongst all imputation strategies. The neuronal types were defined as neurofilament containing (NF), non-peptidergic nociceptors (NP), peptidergic nociceptors (PEP), and tyrosine hydroxylase containing (TH).

**Figure 5:**
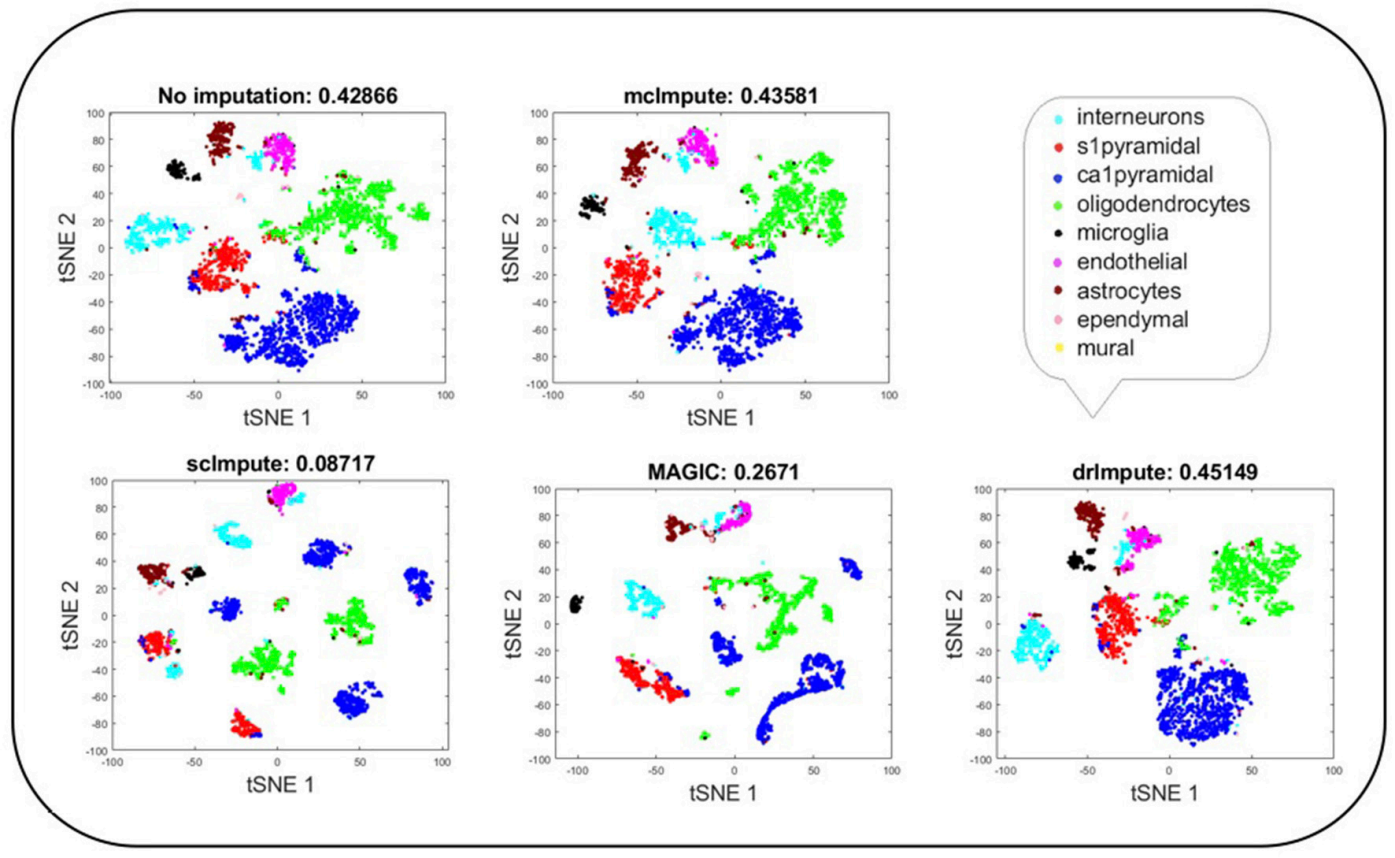
Plot showing t-SNE visualization and average silhouette values for Zeisel dataset before and after imputation. Both mcImpute and drImpute bring brain cells closer, at the same time maintaining the structure of gene-expressions.

Representing scRNA-seq data visually would involve reducing the gene-expression matrix to a lower dimensional space and then plotting each cell transcriptome in that reduced two or three-dimensional space. Two well-known techniques for dimensionality reduction are PCA and t-SNE (Holland, 2008; Maaten and Hinton, 2008). It has been shown that t-Distributed Stochastic Neighbor Embedding (t-SNE) is particularly well suited and effective for the visualization of high-dimensional datasets (Liu et al., 2017). So, we use t-SNE (Figures 4, 5) on Usoskin and Zeisel expression matrices to explore the performance of dimensionality reduction, both without and with imputation. The cells are visualized in 2-dimensional space, coloring each subpopulation by its annotated group, both before and after imputation. To quantify the groupings of cell transcriptomes, we use an unsupervised clustering quality metric, silhouette index. The average silhouette values for each method have been shown in the plot titles (Figures 4, 5 and Figures S3, S4).

T-SNE analysis depicts that mcImpute brings all four groups of mouse neural cells from Usoskin dataset closest to each other in comparison to other methods and performs fairly well, competing with drImpute on Zeisel dataset too.

### 2.7. Improvement in Distribution of Genes

**Figure 6:**
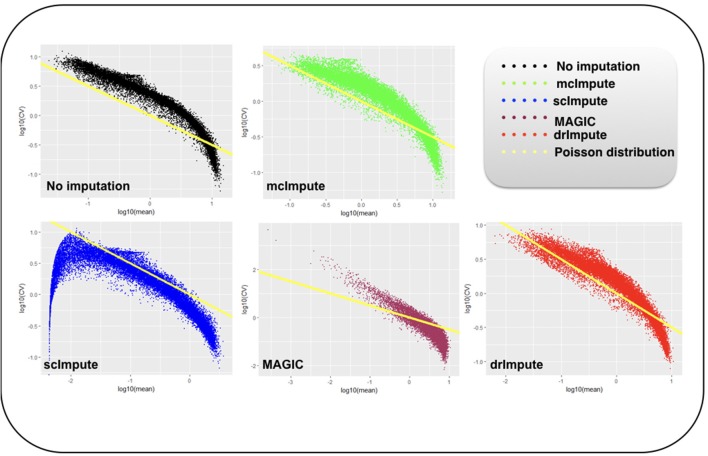
Plot showing log_10_(CV) vs. log_10_(mean) relationship between genes for Preimplantation dataset before and after imputation.

**Figure 7:**
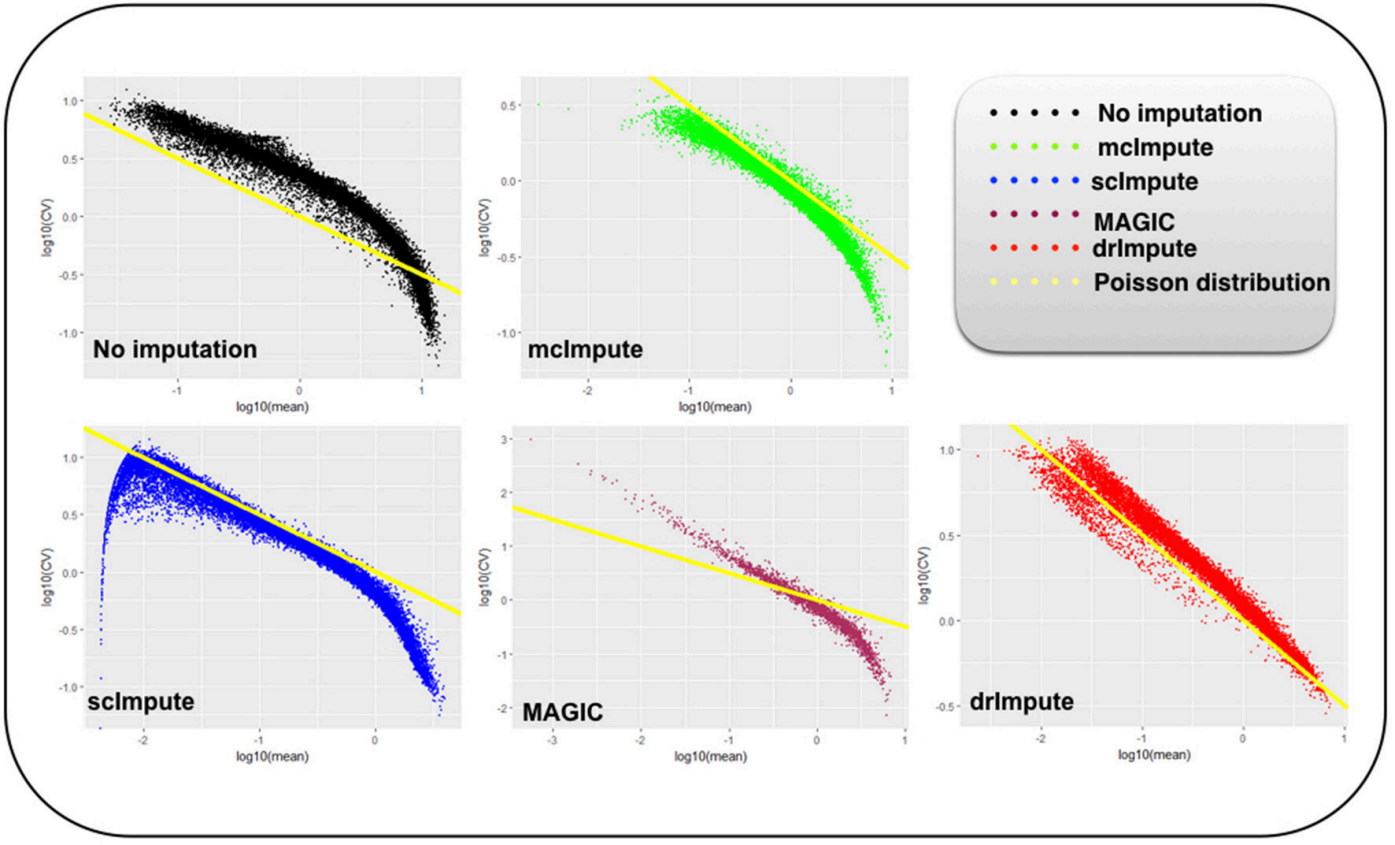
Plot showing log_10_(CV) vs log_10_(mean) relationship between genes for Usoskin dataset before and after imputation.

It has been shown that for single-cell gene expression data, in the ideal condition all genes should obey *CV* = *mean*^−1/2^ (Klein et al., 2015) (CV: coefficient of variation), following a Poisson distribution as depicted by the green diagonal line (Figures 6, 7). This is because individual transcripts are sampled from a pool of available transcripts for CEL-Seq. This accounts for technical noise component which obeys Poissonian statistics (Grün et al., 2014), and thus the CV is inversely proportional to the square root of the mean. Since this result has only been shown for single-cell data with transcript numbers, this experiment has not been analyzed for Jurkat-293T and Zeisel datasets for which the individual RNA molecules were counted using unique molecular identifiers (UMIs).

We model CV as a function of mean expression for all genes to analyze how various imputation methods affect the relationship between them. The results (Figures 6, 7) show that both mcImpute and drImpute succeed to restore the relationship between CV and mean to a great extent (improving the dependency of the CV on the mean expression level to be more consistent with Poissonian sampling noise), while others do not.

## 3. Discussion

Single-cell RNA seq technologies have opened up numerous possibilities for analysis at the single-cell resolution. But, low amount of starting RNA is a major limitation of the technology which results in frequent missing of transcripts in the reverse transcription step (dropout events). This dropout problem in single-cell RNA-seq data makes the expression matrix highly sparse; which in turn hinders the downstream analysis.

To overcome the dropout problem in single-cell data, we take motivation from various areas of applied sciences (including computer vision Tomasi and Kanade, 1992, control Mesbahi and Papavassilopoulos, 1997, machine learning Abernethy et al., 2006; Amit et al., 2007; Argyriou et al., 2007, etc) where recovery of an unknown low-rank matrix from very limited information is of interest. The problem is akin to that of recommendation systems (e.g. in Netflix movie recommendations and Amazon product recommendations) (Bell and Koren, 2007; Bennett and Lanning, 2007; SIGKDD, 2007), where there is a database of ratings given by users to movies/products. Since the users typically rate only a small subset of items, not all the ratings are available; which makes the user-movie rating matrix sparse. Also, the matrix is assumed to be of low-rank because there are not too many independent parameters on which the users generally rate the movie. The objective is to estimate the ratings of all the users on all the movies. If the new movie rating predictions can be done accurately, recommendation accuracy increases. There is a pretty straightforward link between both the Netflix problem and dropout problems. Therefore, imputation to single-cell expression matrix can be efficiently performed by Low-rank approximation. (Koren et al., 2009; Majumdar and Ward, 2011).

One could argue about the low-rank origin of the gene expression data. It should be noted that numerous studies have suggested that genes do not work in isolation (Staiger et al., 2013), but as part of a complex regulatory network (Silver et al., 2013). This inter-dependency has been analyzed in the form of associated network structures (Xiong et al., 2005; Gill et al., 2010) and is best reflected by the gene-gene correlations (Weckwerth et al., 2004; Klebanov and Yakovlev, 2007; Reynier et al., 2011; Najafov and Najafov, 2018). It is so believed that such high levels of correlation are caused by sharing of regulatory programs among different genes (Ye et al., 2013). Also, it has previously been shown that a small number of interdependent biophysical functions trigger the functioning of transcription factors, which in turns influence the expression levels of genes, resulting in a highly correlated data matrix (Kapur et al., 2016). On the other hand, cells coming from same tissue source also lie on differential grades of the variability of a limited number of phenotypic characteristics. Therefore, it is just to assume that the gene expression values lie on a low-dimensional linear subspace and the data matrix thus formed may well be thought as a low-rank matrix.

We attempt to give another mathematical justification on the Low-rank assumption of the gene-expression in Figure S2 by showing that the maximum information of the expression-data is held in its first few singular values; hence the rank of the expression matrix (number of non-zero singular values) should be low.

In specific, we used Nuclear Norm-based Matrix Completion for imputing single-cell RNA seq data. The algorithm models the single-cell gene expression as a low-rank matrix and recovers the full gene expression from partial information by thresholding the singular values of expression matrix iteratively. The recovery process sprouts-in appropriate expressions in place of dropouts; keeping the biologically silent expression values intact.

Apart from taking care of biologically silent genes, the proposed algorithm performs competitively with the state-of-the-art methods in improving the clustering accuracy of cells, identifying differentially expressed genes, enhancing cell type separability, improving the dimensionality reduction, etc.

Our method is particularly suitable for single-cell data since it does not assume anything about the statistical property of the expression or the dropouts and can be seamlessly incorporated into the single-cell analysis pipeline. We have also demonstrated that our method clearly distinguishes between biological and technical silencing.

The algorithm has some scope of improvement when it comes to handling scRNA– seq datasets with large sample sizes. As can be seen in Table S5, the running time of our algorithm is comparatively more than that of MAGIC and drImpute; although much less than that of scImpute.

## 4. Data and Methods

### 4.1. Dataset Description

We used five scRNA-seq datasets from four different studies for performing various experiments (Table S7).

- **Jurkat-293T:** This dataset contains expression profiles of Jurkat and 293T cells, mixed *in vitro* at equal proportions (50:50). All ~ 3,300 cells of this data are annotated based on the expressions of cell-type specific markers (Zheng et al., 2017). Cells expressing CD3D are assigned Jurkat, while those expressing XIST are assigned 293T. This dataset is also available at 10x Genomics website (https://support.10xgenomics.com/single-cell-gene-expression/datasets/1.1.0/jurkat:293t_50:50).
- **Preimplantation:** This is an scRNA-seq data of mouse preimplantation embryos. It contains expression profiles of ~ 300 cells from zygote, early 2-cell stage, middle 2-cell stage, late 2-cell stage, 4-cell stage, 8-cell stage, 16-cell stage, early blastocyst, middle blastocyst, and late blastocyst stages. The first generation of mouse strain crosses was used for studying monoallelic expression. We downloaded the count data from Gene Expression Omnibus (GSE45719) (Yan et al., 2013).
- **Zeisel:** Quantitative single-cell RNAseq has been used to classify cells in the mouse somatosensory cortex (S1) and hippocampal CA1 region based on 3005 single cell transcriptomes (Zeisel et al., 2015). Individual RNA molecules were counted using unique molecular identifiers (UMIs) and confirmed by single-molecule RNA fluorescence *in situ* hybridization (FISH). A divisive biclustering method based on sorting points into neighborhoods (SPIN) was used to discover molecularly distinct, 9 major classes of cells. Raw data is available under the accession number GSE60361.
- **Usoskin:** This data of mouse neurons (Usoskin et al., 2015) was obtained by performing RNA-Seq on 799 dissociated single cells dissected from the mouse lumbar dorsal root ganglion (DRG) distributed over a total of nine 96-well plates. After Principal component analysis (PCA) of expression magnitudes across all cells and genes, 622 cells were classified as neurons, 68 cells had an ambiguous assignment and 109 cells were non-neuronal. We take into account the 622 neuronal clusters of mouse lumbar DRG- neurofilament containing (NF), non-peptidergic nociceptors (NP), peptidergic nociceptors (PEP), and tyrosine hydroxylase containing (TH). RPM normalized counts are available under the accession number GSE59739.
- **Trapnell:** This is an scRNA-seq data of primary human myoblasts (Trapnell et al., 2014). Differentiating myoblasts were cultured and cells were dissociated and individually captured at 24-h intervals. 50–100 cells at each of the four time points were captured following serum switch using the Fluidigm C1 microfluidic system. This data is available at Gene Expression Omnibus under the accession number GSE52529. Of note, this dataset has been used for the experiments which require the Bulk-counterpart of the gene-expression data i.e., “Dropout vs true-zeros” and “Differential genes prediction.”

### 4.2. Data Preprocessing

Steps involved in preprocessing of raw scRNA-seq data are enumerated below.

- **Data filtering:** It is ensured that data has no bad cells and if a gene was detected with ≥3 reads in at least 3 cells we considered it expressed. We ignored the remaining genes.
- **Library-size Normalization:** Expression matrices were normalized by first dividing each read count by the total counts in each cell, and then by multiplying with the median of the total read counts across cells.
- **Log Normalization:** A copy of the matrices were log_2_ transformed following the addition of 1 as pseudo-count.
- **Imputation:** Further, log transformed expression matrix was used as input to mcImpute. The algorithm returns imputed log transformed matrix, normalized matrix (after applying reverse of log operation on imputed log-transformed expressions), and the count matrix after imputation.

A brief overview of the complete mcImpute pipeline has been shown in Figure 1.

### 4.3. Low-Rank Matrix Completion: Definition

Our problem is to complete a partially observed gene expression matrix *X* where columns represent genes and rows, individual cells. The complete matrix is constituted by the known and the yet unknown values. We can assume that the single cell data that we have acquired, *Y* is a sampled version of the complete expression matrix *X*. Mathematically, this is expressed as,

(1) Y=A(X)

Here *A* is the sub-sampling operator. It is a binary mask that has 0's where the counts of complete expression data *X* have not been observed and 1's where they have been. The values of A are element-wise multiplied to the complete expression matrix X so that Y (the sub-sampled data) is a sparse representation of X and has expression values only at positions where gene expression is observed. Our problem is to recover *X*, given the observations *Y*, and the sub-sampling mask *A*. It is known that *X* is of low-rank.

It should be noted that matrix completion is a well studied framework. In this work, we consider two algorithms for efficient imputation of scRNA-seq expression data: Matrix factorization (Koren et al., 2009) and Nuclear norm minimization?

### 4.4. Matrix Factorization

Matrix factorization is the most straightforward way to address the low-rank matrix completion problem; it has previously been used for finding lower dimensional decompositions of matrices (Lee and Seung, 2001). Say *X* is of dimensions *m* × *n*, but is known to have a rank *r* (<*m, n*). In that case, one can express *X*_*m* × *n*_ as a product of two matrices *U*_*m* × *r*_ and *V*_*r* × *n*_ . Therefore the complete problem (1) can be formulated as,

(2) Y=A(X)=A(UV)

Estimating *U* and *V* from (2) tantamount to recovering X. The two matrices *U* and *V* can be solved by minimizing the *Frobenius* norm of the following cost function.

(3) $$\underset{U,V}{\min}||Y-A(UV)|{|}_{F}^{2}$$

Since this is a bi-linear problem, one cannot guarantee global convergence. However, it usually works in practice. It has been used for solving recommender systems problems (Koren et al., 2009), where (3) was solved using stochastic gradient descent (SGD). SGD is not an efficient techniques and requires tuning of several parameters. In this work, we will solve (3) in a more elegant fashion using Majorization-Minimization (MM) (Sun et al., 2017). The basic MM approach and its geometrical interpretation has been diagrammatically represented (Figure S1). It depicts the solution path for a simple scalar problem but essentially captures the MM idea.

For our given problem, the cost function to be minimized is given as $J\left(X\right)=||Y-A\left(X\right)|{|}_{F}^{2}$; the majorization step basically decouples the problem (from *A*), so that we can solve the optimization problem by solving

(4) $$\underset{U,V}{\min}||B-UV|{|}_{F}^{2}$$

where $B_{k+1}=X_{k}+\frac{1}{a}A^{T}\left(Y-A\left(X_{k}\right)\right)$ at each iteration k. Here, *X*_*k*_ is the matrix at iteration k and *a* is a scalar parameter in the MM algorithm.

This (4) is solved by alternating least squares (Hastie et al., 2015), i.e., while updating *U*, *V* is assumed to be constant and while updating *V*, *U* is assumed to be constant.

(5) $$U_{k}\leftarrow \underset{U}{\min}||B-U_{k-1}V_{k-1}|{|}_{F}^{2}$$

(6) $$V_{k}\leftarrow \underset{V}{\min}||B-U_{k}V_{k-1}|{|}_{F}^{2}$$

Since the log-transformed input (with pseudo count added) expressions would never be negative, we have imposed a non-negativity constraint on the recovered matrix X, so that it does not contain any negative values.

The matrix factorization algorithm has been summarized in Algorithm 1. The initialization of factor V is done by keeping *r* right singular vectors of X in V obtained by performing singular value decomposition (SVD) of X, where *r* is the approximate rank of the expression matrix to be recovered.

**Algorithm 1:**
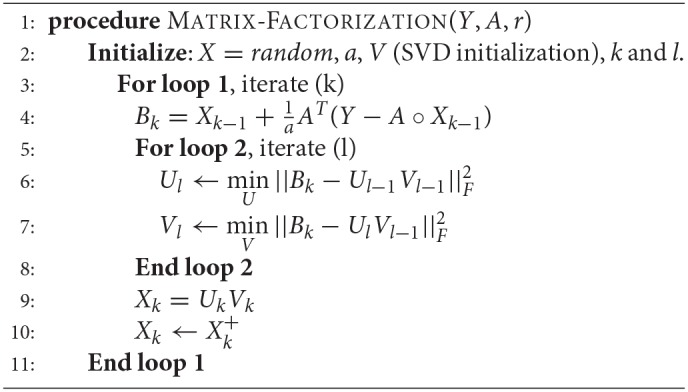
Matrix completion using matrix factorization

### 4.5. Nuclear Norm Minimization

The problem depicted in (3) is non-convex. Hence, there is no guarantee for global convergence. Also one needs to know the approximate rank of the matrix *X* in order to solve it, which is unknown in this case. To combat this issues, researchers in applied mathematics and signal processing proposed an alternative solution. They would directly solve the original problem (1) with a constraint that the solution is of low-rank. This is mathematically expressed as,

(7) $$\underset{X}{\min}rank(X)\text{ such that }Y=A(X)$$

However, this turns out to be NP hard problem with doubly exponential complexity. Therefore, studies in matrix completion (Candes and Recht, 2009; Candès and Tao, 2010) proposed relaxing the NP hard rank minimization problem to its closest convex surrogate: nuclear norm minimization.

(8) $$\underset{X}{\min}||X|{|}_{*}\text{ such that }Y=A(X)$$

Here ||.||_*_ is the nuclear norm and is defined as the sum of singular values of data matrix *X*. It is the *l*_1_ norm of the vector of singular values of X and is the tightest convex relaxation of the rank of matrix, and therefore its ideal replacement.

This is a semi-definite programming (SDP) problem. Usually its relaxed version (Quadratic Program) is solved (Candès and Plan, 2010) with the unconstrained Lagrangian version.

(9) $$\underset{X}{\min}||Y-A(X)|{|}_{F}^{2}+\lambda ||X|{|}_{*}$$

Here, ||.||_*_ is the nuclear norm and λ is called the Lagrange multiplier. The problem (9) does not have a closed form solution and needs to be solved iteratively.

To solve (9), we invoke MM once more. Here $J\left(X\right)=||Y-A\left(X\right)|{|}_{F}^{2}+\lambda ||X|{|}_{*}$, we can express (9) in the following fashion in every iteration *k*

(10) $$\underset{X}{\min}||B-X|{|}_{F}^{2}+\lambda ||X|{|}_{*}$$

where $B_{k+1}=X_{k}+\frac{1}{a}A^{T}\left(Y-A\left(X_{k}\right)\right)$.

Using the inequality ||*Z*_1_−*Z*_2_||_*F*_≥||*s*_1_−*s*_2_||_2_ , where *s*_1_ and *s*_2_ are singular values of the matrices *Z*_1_ and *Z*_2_ respective, we can solve the following instead of solving the minimization problem (10).

(11) $$\underset{s_{x}}{\min}||s_{B}-s_{X}|{|}_{2}^{2}+\lambda ||s_{X}|{|}_{1}$$

Here, *s*_*B*_ and *s*_*X*_ are the singular values of *B* and *X*, respectively and ||*s*_*X*_||_1_ is the *l*_1_ norm or the sum of absolute values of *s*_*X*_. It has been shown that problem (10) is minimized by soft thresholding the singular values with threshold λ/2. The optimal update is given by

(12) $$s_{X}=\{\begin{array}{l} s_{B}+\lambda /2\text{when }s_{B}\leq -\lambda /2\\ 0\text{ when}|s_{B}|\leq \lambda /2\\ s_{B}-\lambda /2\text{ when }s_{B}\geq \lambda /2 \end{array}$$

or more compactly by

(13) $$s_{X}=soft(s_{B},\lambda /2)=sign(s_{B})max(0,|s_{B}|-\lambda /2)$$

We found that the algorithm is robust to values of λ as long as as it is reasonably small (< 0.01).

Here too, we have imposed the non-negativity constraint on *X* since expressions cannot be smaller than zero. The Nuclear Norm Minimization algorithm has been depicted in Algorithm 2.

**Algorithm 2:**
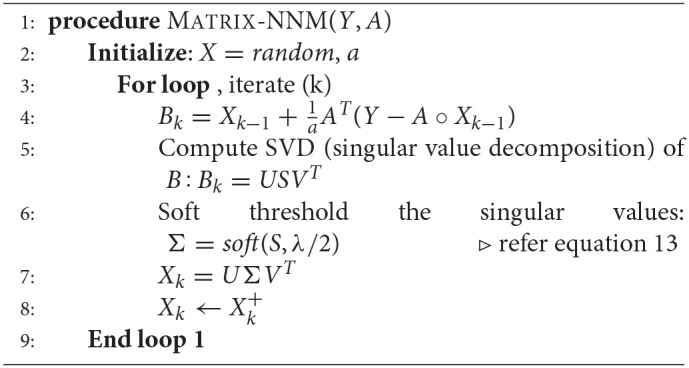
Matrix completion via nuclear norm minimization

## 5. Conclusion

As an inevitable consequence of a steep decline in single cell library depth, dropout rates in scRNA-seq data have skyrocketed. This works as a confounding factor (Hicks et al., 2015), thereby hindering cell clustering and further downstream analyses. A good imputation strategy would handle the Dropouts problem gracefully and thereby has the potential to facilitate the discovery of new rare cell subtypes within seemingly similar cells. This, in turn, can be helpful for characterizing cancer heterogeneity and understanding the dynamics of transcriptional changes during development. The proposed mcImpute algorithm, without making any assumption about the expression data distribution, recovers dropouts by simultaneously retaining the true zero counts and shows comparable performance on a number of measures including clustering accuracy, cell type separability, differential gene prediction, cell visualization, gene distribution, etc.

We believe that McImpute, by far is the most intuitive way of catering the dropouts problem. It can seamlessly be integrated and serve as a key component in single-cell RNA seq pipeline.

Currently, imputation and clustering are together a piecemeal two-step process—imputation followed by clustering. In the future, we would like to incorporate both clustering and imputation as a joint optimization problem.

## 6. Software

The source code of mcImpute is shared at https://github.com/aanchalMongia/McImpute_scRNAseq.

## Data Availability Statement

The details of datasets for this study has been given in section 4.

## Author Contributions

DS and AnM led the study, contributed to the statistical analysis and design of the experiments. AaM analyzed and interpreted the scRNA-seq data and performed the experiments. All authors read and reviewed the manuscript.

### Conflict of Interest Statement

The authors declare that the research was conducted in the absence of any commercial or financial relationships that could be construed as a potential conflict of interest.
