# Supplementary material for: McImpute: Matrix Completion Based Imputation for Single Cell RNA-seq Data
Source: Front Genet. 2019 Jan 29;10:9. doi: 10.3389/fgene.2019.00009 (PMC6361810; doi:10.3389/fgene.2019.00009)
Supplement: Supplementary file 1 [file Data_Sheet_1.PDF]

McImpute: Matrix completion based imputation for single cell RNA-seq data

SUPPLEMENTARY MATERIAL

Aanchal Mongia[3], **Debarka Sengupta**[2,3*], and **Angshul Majumdar** [1]

[1] *Dept. of Electronics and Communications Engineering, IIIT - Delhi, 110020*
[2] *Center for Comp. Biol., IIIT - Delhi, 110020*
[3] *Dept. of Comp. Sc. and Eng., IIIT - Delhi, 110020*

[*] *Corresponding author: {debarka,angshul}@iiitd.ac.in*

Compiled December 8, 2018

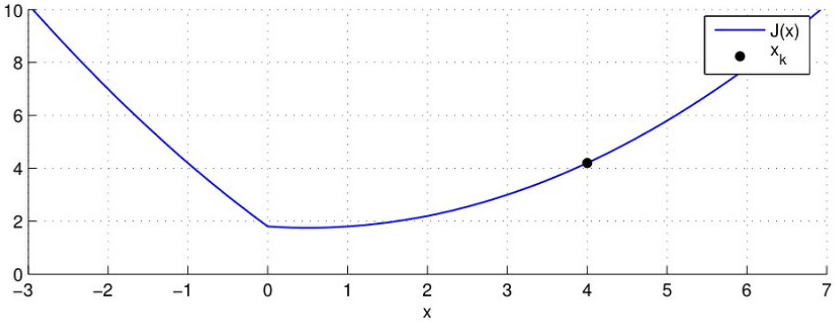

(a) Function J(x) to be minimized

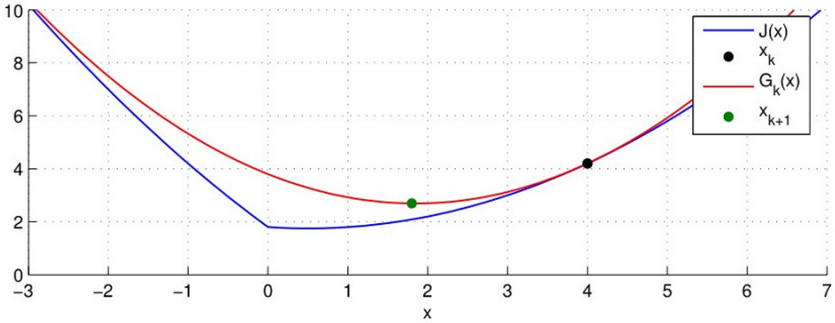

(b) One iteration of MM

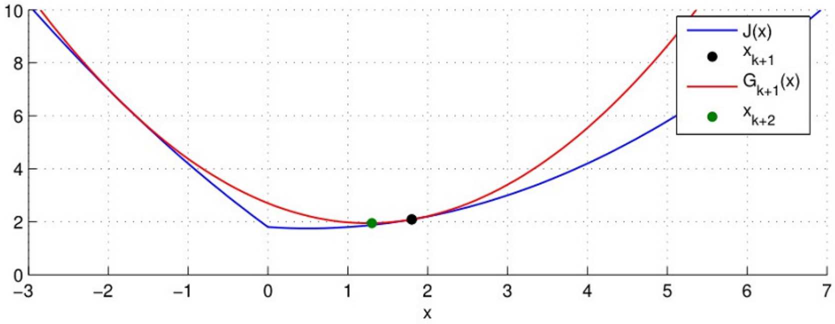

(c) Subsequent iteration

Fig. S1. Majorization Minimization - Schematic Diagram: Let, $J(x)$ be the function to be minimized. Start with an initial point (at $k = 0$) x_k (**sub figure (a)**). A smooth function $G_k(x)$ is constructed through x_k which has a higher value than $J(x)$ for all values of x apart from x_k, at which the values are the same. This is the Majorization step. The function $G_k(x)$ is constructed such that it is smooth and easy to minimize. At each step, minimize $G_k(x)$ to obtain the next iterate x_{k+1} (**sub figure (b)**). A new $G_{k+1}(x)$ is constructed through x_{k+1} which is now minimized to obtain the next iterate x_{k+2}. As can be seen, the solution at every iteration gets closer to the actual solution.

DATASET	BINS									
	0	0-2	2-4	4-6	6-10	10-20	20-30	30-50	50-100	100-500
No imputation	0.9942	0.9679	0.9422	0.9321	0.9334	0.8905	0.8745	0.8594	0.7932	0.5678
mcImpute	0.9893	0.9367	0.8712	0.8294	0.8165	0.6726	0.5836	0.5000	0.3493	0.0958
scImpute	0.9881	0.9273	0.8405	0.8052	0.7964	0.6476	0.5912	0.5094	0.3868	0.1503
MAGIC	0.9989	0.9911	0.9819	0.9825 0.	9805	0.9419	0.9285	0.9220	0.8213	0.4289

Table S1. Separation of "true zeros" from dropouts: Fraction of zeros (values between 0 and 0.5) in single cell expression matrix against the median bulk expression. The genes are divided into 10 bins based on median bulk genes expression (first bin corresponds to zero expression genes)

DATASET	METHOD				
	No Imputation	mcImpute	scImpute	MAGIC	drImpute
Jurkat-293T	0.987	0.986	0.968	0.982	0.910
Preimplantation	0.328	0.367	0.431	0.373	0.199
Usoskin	0.297	0.317	0.274	0.244	0.018
Zeisel	0.357	0.401	0.364	0.377	0.320

Table S2. Clustering accuracy: Average Adjusted Rand Index values (on 100 runs of PCA followed by k-means) measuring the correspondence between the k-means predicted clusters and the prior annotations.

Sub-sampling ratio	NMSE		RMSE		MAE	
	MF	NNM	MF	NNM	MF	NNM
50%	0.6876	0.6569	1.3587	1.2970	0.8371	0.7589
60%	0.6726	0.6365	1.3281	1.2585	0.8099	0.7430
70%	0.6625	0.6237	1.3080	1.2330	0.7910	0.7307
80%	0.6569	0.6155	1.2968	1.2169	0.7781	0.7228
90%	0.6518	0.6104	1.2880	1.2063	0.7692	0.7159

Table S3. Matrix recovery error: Comparison of NMSE, RMSE and MAE between recovered matrices and unmasked Usoskin data using Nuclear Norm Minimization (NNM) and Matrix Factorization (MF) algorithms at hidden/masked positions

DATASET		METHOD				
		No Imputation	mcImpute	scImpute	MAGIC	drImpute
JURKAT-293T	Intra Jurkat	0.5018	0.7076	0.9315	0.9923	0.8768
	Intra 293T	0.5029	0.7675	0.9125	0.9893	0.9070
	Inter	0.4402	0.5552	0.8119	0.8641	0.7185
	cell type separability	0.0621	0.1823	0.1101	0.1267	0.1734
PREIMPLANTATION	Intra 8cell	0.5454	0.8670	0.6504	0.9972	0.7823
	Intra BXC	0.7669	0.9022	0.8989	0.9989	0.8850
	Inter	0.3888	0.5252	0.5506	0.9440	0.6051
	cell type separability	0.2674	0.3594	0.2241	0.0540	0.2286
USOSKIN	Intra NP	0.4441	0.7945	0.8792	0.9974	0.9093
	Intra NF	0.5265	0.7783	0.8931	0.9974	0.8773
	Inter	0.4346	0.7091	0.8347	0.9740	0.7946
	cell type separability	0.0507	0.0773	0.0515	0.0234	0.0987
ZEISEL	Intra-S1pyramidal	0.4867	0.7483	0.8750	0.9914	0.8867
	Intra-ependymal	0.3574	0.6051	0.7700	0.9765	0.8106
	Inter	0.2723	0.4893	0.7496	0.9249	0.8042
	cell type separability	0.1498	0.1874	0.0729	0.0590	0.0444

Table S4. CTS scores: Cell type separability (CTS) for any 2 randomly chosen cell groups from each dataset.

DATASET	METHOD			
	mcImpute	scImpute	MAGIC	drImpute
Jurkat-293T	60.38	338.80	0.97	83.50
Preimplantation	1.79	6.79	0.62	0.52
Usoskin	3.35	6.62	0.07	1.12
Zeisel	62.09	90.40	0.36	40.10

Table S5. Time taken: Time (in minutes) taken by each imputation algorithm on various datasets used.

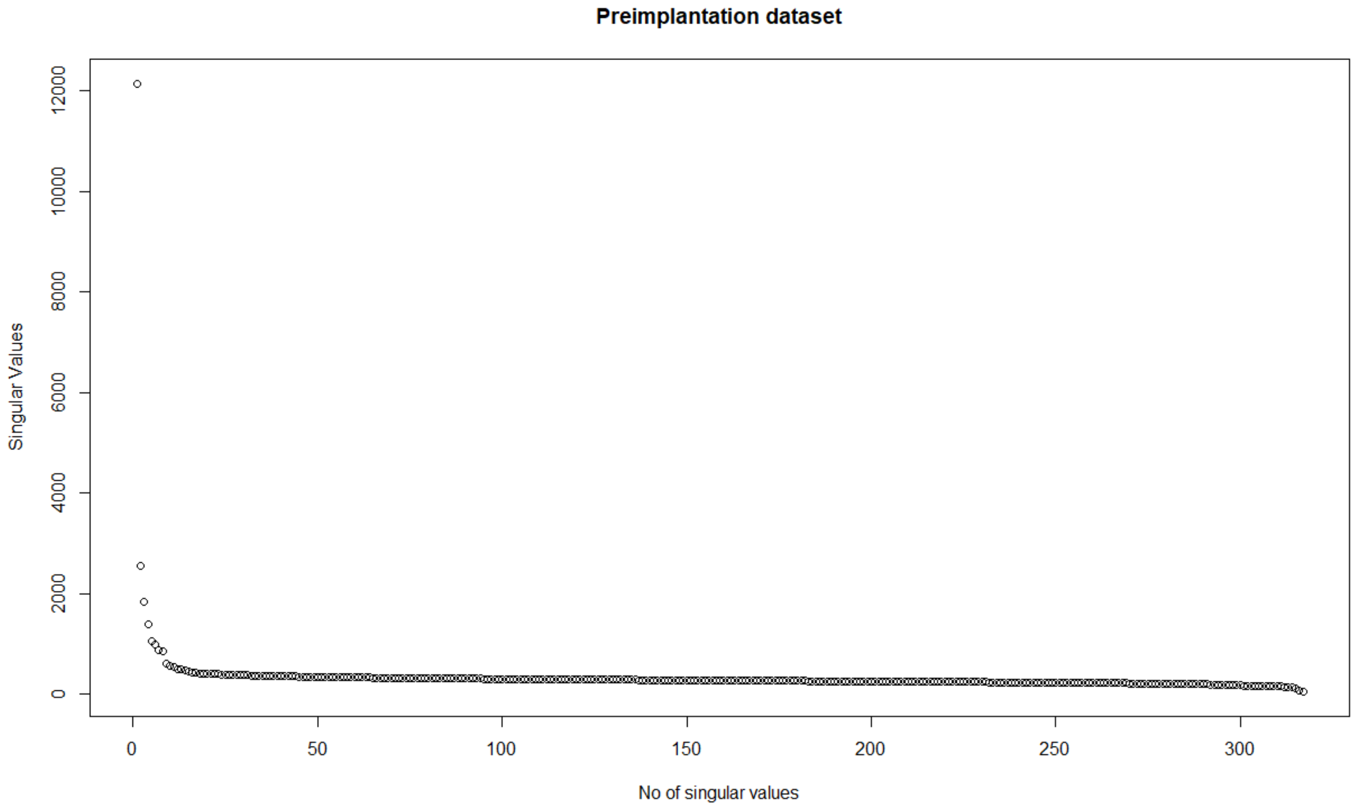

Fig. S2. Decay of singular values: The given plot of singular values of Preimplantation dataset shows that the maximum energy of the data lies in the first few singular values, plotted in decreasing order of their value. Hence, it can be argued that the number of singular values retaining the actual information about the expression values is very low, thereby justifying the low-rank assumption on the single-cell gene-expression dataset.

	PREVIOUS METHODS		
NAME	**scImpute**	**MAGIC**	**drImpute**
CORE CONCEPT	Borrow information from similar cells	Markov-Affinity matrix	Cell-clustering +averaging
DROPOUT DISCRIMINATION	Yes	No	Yes
APPROACH	Gammma-Normal mixture model	—–	Down-sampling based simulation

Table S6. Previous methods: Summary of previous methods proposed for imputation of the scRNA-seq data sets.

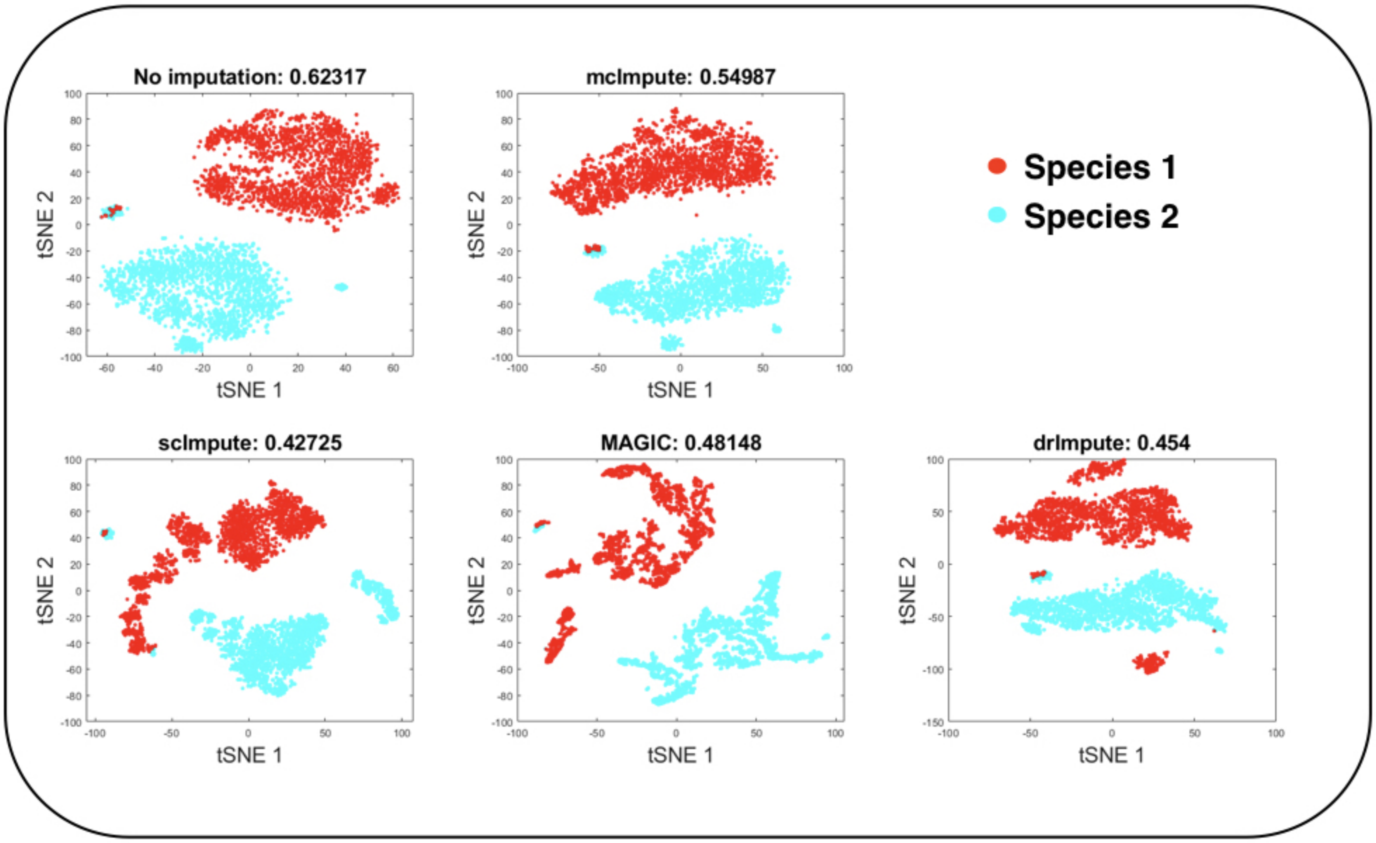

Fig. S3. Plot showing t-SNE visualization and average silhouette values for Jurkat-293T dataset before and after imputation. The striking difference between Jurkat and 293T cell types made them trivially separable.

DATASETS USED	#Cell-types	#Cells	Cell-Annotation information	Availability	Reference
Jurkat-293T	2	3388	Cell populations annoated based on cell-type specific markers.	[1]	[2]
Preimplantation	13	317	Stages of human preimplantation development	GSE45719	[3]
Usoskin	4	622	Clusters of mouse lumbar dorsal root ganglion (DRG)	GSE59739	[4]
Zeisel	9	3005	Cell populations from mouse cortex and hippocampus	GSE60361	[5]
Trapnell	4	384	Time points in differentiated primary human myoblasts	GSE52529	[6]

Table S7. Datasets used: Summary of the scRNA-seq datasets used in the study.

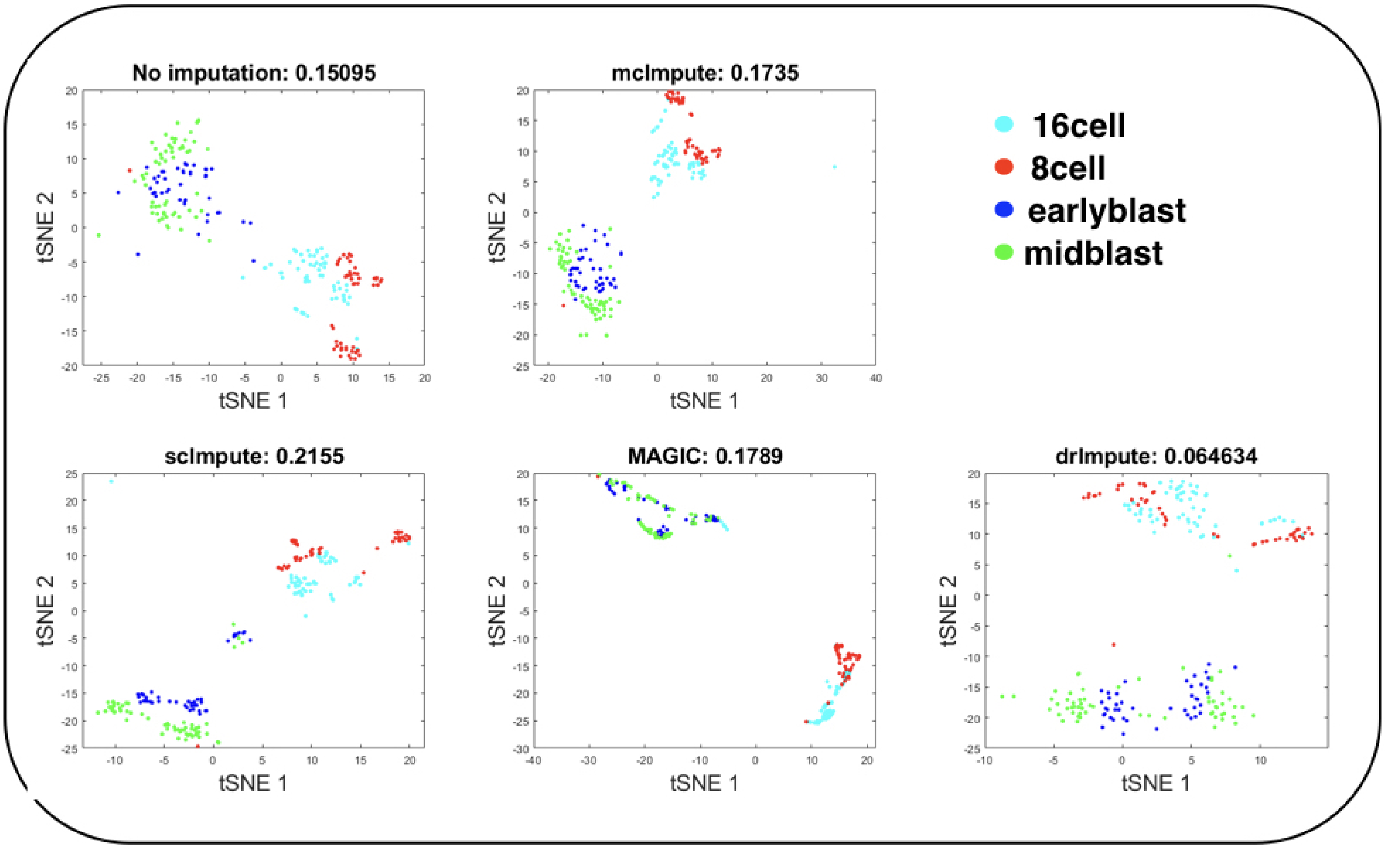

Fig. S4. Plot showing t-SNE visualization and average silhouette values for Preimplantation dataset before and after imputation. Out of the 13 cell-stages of human preimplantation development, we display the cells corresponding to the four most prominent types which are in majority. Visually, both mcImpute and scImpute bring the cells of each type closer to each other.

APPROACHES	DATASETS					PREVIOUS METHODS		
	Jurkat-293T	Preimplantation	Usoskin	Zeisel	Trapnell	scImpute	MAGIC	drImpute
Dropout vs true-zeros	×	×	×	×	✓	✓	✓	×
Matrix recovery	×	×	✓	×	×	×	×	×
Clustering	✓	✓	✓	✓	×	✓	✓	✓
Differential genes prediction	×	×	×	×	✓	✓	✓	✓
Cell type separability	✓	✓	✓	✓	×	✓	✓	✓
Cell visualization	✓	✓	✓	✓	×	✓	✓	✓
Gene distribution	×	✓	✓	×	×	✓	✓	✓

Table S8. Datasets Vs Approaches: The table summarizes the datasets used and the previous methods with which mcImpute has been compared with; for every approach/experiment used in the analysis.

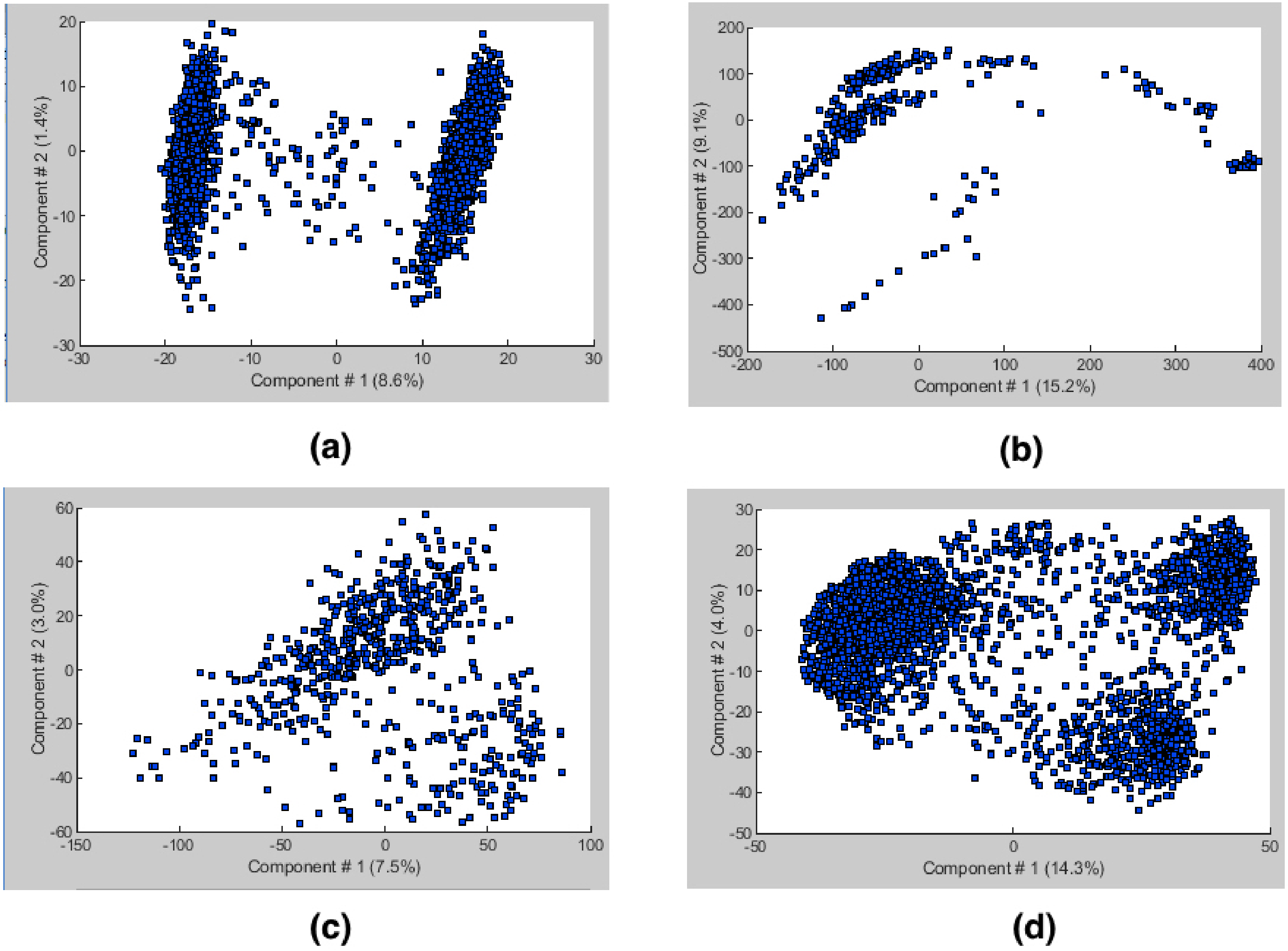

Fig. S5. Percentage of explained variance by first two principal components: Principal component analyses (PCA) was applied on all the datasets (a) Jurkat-293T (b) Preimplantation (c) Usoskin and (d) Zeisel. The percentage of total variance explained by each of the first few principal components were analyzed and the components were selected (for input to clustering algorithm) such that at least 10 % of total variance is accounted for. This explains the rationale for selecting the first 2 principal components for the clustering analysis (section 2.2).

REFERENCES

1. "10x Genomics," https://support.10xgenomics.com/single-cell-gene-expression/datasets.
2. G. X. Zheng, J. M. Terry, P. Belgrader, P. Ryvkin, Z. W. Bent, R. Wilson, S. B. Ziraldo, T. D. Wheeler, G. P. McDermott, J. Zhu *et al.*, "Massively parallel digital transcriptional profiling of single cells," Nat. communications **8**, 14049 (2017).
3. L. Yan, M. Yang, H. Guo, L. Yang, J. Wu, R. Li, P. Liu, Y. Lian, X. Zheng, J. Yan *et al.*, "Single-cell rna-seq profiling of human preimplantation embryos and embryonic stem cells," Nat. structural & molecular biology **20**, 1131–1139 (2013).
4. D. Usoskin, A. Furlan, S. Islam, H. Abdo, P. Lönnerberg, D. Lou, J. Hjerling-Leffler, J. Haeggström, O. Kharchenko, P. V. Kharchenko *et al.*, "Unbiased classification of sensory neuron types by large-scale single-cell rna sequencing," Nat. neuroscience **18**, 145 (2015).
5. A. Zeisel, A. B. Muñoz-Manchado, S. Codeluppi, P. Lönnerberg, G. La Manno, A. Juréus, S. Marques, H. Munguba, L. He, C. Betsholtz, C. Rolny, G. Castelo-Branco, J. Hjerling-Leffler, and S. Linnarsson, "Cell types in the mouse cortex and hippocampus revealed by single-cell rna-seq," Science **347**, 1138–1142 (2015).
6. C. Trapnell, D. Cacchiarelli, J. Grimsby, P. Pokharel, S. Li, M. Morse, N. J. Lennon, K. J. Livak, T. S. Mikkelsen, and J. L. Rinn, "Pseudo-temporal ordering of individual cells reveals dynamics and regulators of cell fate decisions," Nat. biotechnology **32**, 381 (2014).
